# Creation and Psychometric Validation of “Nursing Competencies Questionnaire on Older People’s Environmental Health (NCQ‐OPEH)” in Nurses and Nursing Students

**DOI:** 10.1155/nrp/1783950

**Published:** 2026-07-24

**Authors:** Eva M. Montoro-Ramírez, Laura Parra-Anguita, Carmen Álvarez-Nieto, Isabel M. López-Medina

**Affiliations:** ^1^ Department of Nursing, Faculty of Health Sciences, Nursing Research and Innovation in Health Care Research Group (CuiDsalud CTS 464), University of Jaen, Jaen, Spain, ujaen.es

**Keywords:** aged, climate change, clinical competence, nurses, nursing, students, validation study

## Abstract

**Aim:**

To develop and psychometrically validate an instrument to assess the competencies (knowledge, skills, and attitudes) of nurses and nursing students regarding the effects of climate change on the health of older people.

**Methods:**

Cross‐sectional descriptive study of questionnaire construction and validation developed in four phases: creation and elaboration of the initial items, content validation by an expert panel, pilot test, and psychometric validation. A tool was developed consisting of a knowledge questionnaire, a skills scale, and an attitude scale. Data were collected from a convenience sample of 708 individuals (210 nurses and 498 nursing students) between January and April 2024. Content validation was carried out by consulting a panel of 13 experts and a pilot test. Psychometric validation was carried out using Item Response Theory, specifically the Rasch model for the knowledge questionnaire and Andrich’s rating scale model for the skills and attitude scales. The STROBE checklist for cross‐sectional studies was followed.

**Results:**

Reliability for the set of items and for individuals was excellent (0.99 and 0.90, respectively). Item separation index was above 2 in all three parts of the instrument, although somewhat more limited for people. Internal consistency was acceptable in the knowledge questionnaire (11 items, Cronbach’s *α* 0.68) and excellent in the skills (13 items, Cronbach’s *α* 0.91) and attitudes (11 items, Cronbach’s *α* 0.93) scales.

**Conclusion:**

Nursing Competencies Questionnaire on Older People’s Environmental Health is a useful and reliable instrument for measuring knowledge, skills, and attitudes in nurses and nursing students.

**Patient or Public Contributions**
• Nurses and nursing students participated in data collection. Their contributions included completing the questionnaire to be validated, both in the pilot test and in the validation phase, ensuring that the research had the appropriate number of participants to perform psychometric validation reliably.

## 1. Introduction

There is growing concern today about how climate change will affect human health and, more specifically, the most vulnerable population, such as those over 65 years of age. Climate change consequences affect this population group more intensely. It is expected that in older adults, the mortality rate related to adverse climate effects will increase by 145% in the coming decades [1]. In the same way, the mental health of this population is also affected by climate change, either by generating new problems or exacerbating chronic conditions [2]. Furthermore, the number of individuals over 65 years of age is growing, resulting in the aging of the world population, an unprecedented phenomenon that is accelerating in recent decades [3]. Two important problems therefore come together: climate change and rapid demographic aging of the world’s population, which will lead to greater health demand from this age group exposed to adverse climatic effects [1].

Nurses are in a unique position to address the effects of climate change on the health of older adults. As climate change accelerates, so will the need to treat climate‐related illnesses in this population group [4]. Nursing professionals must therefore have the necessary environmental competencies to, for example, be able to identify health problems related to climate change; readjust care plans according to the increased climate risk of exposed older people; identify individuals with greater exposure or sensitivity to the effects of climate change; be informed about early warnings of meteorological phenomena and the prediction of outbreaks of climate‐related diseases (epidemiological surveillance) [4–6]. It is also essential that during their university education, nursing students receive education for sustainable development (ESD) through the incorporation of skills to promote planetary health. It is necessary to instruct nursing students in the knowledge, skills, and attitudes required for the development of actions aimed at finding solutions to combat climate change and its effects on the environmental health of the older population [7].

The KSA (Knowledge, Skills, Attitude) conceptual framework proposed by Guzman et al. [8] suggests that these competencies should be considered more than the simple acquisition of knowledge regarding the effects of climate change on the health of this vulnerable population. Nurses and nursing students must also be able to demonstrate the skills required for their healthcare and the appropriate attitude to deliver sustainable care [9, 10]. The safety of older patients depends directly on the professional’s capacity to respond. An evaluation tool based on the KSA model provides a mechanism for self‐assessment and professional development. Furthermore, it also acts as an indicator of quality of care since if the nurse is able to integrate these three components, resilient care is ensured in an increasingly unpredictable climate [9]. For nursing students, the KSA model allows them to integrate its components to achieve meaningful learning, avoiding an isolated approach to each of them [10].

There are several instruments that measure, in a separate manner, the knowledge, skills, or attitudes of nurses and nursing students about the effect of climate change on the health of the general population [11–13]. Regarding populations that are particularly vulnerable to these effects, there is only one validated tool to measure the knowledge and skills of nursing students about children’s environmental health [14]. However, there is no tool for measuring these competencies on older people’s environmental health. The aim of this research was to create and psychometrically validate an instrument that allows the evaluation of competencies (knowledge, skills, and attitudes) in nursing professionals and nurse students regarding the effects of climate change on the health of older people.

## 2. Methods

### 2.1. Design

A descriptive cross‐sectional study was carried out for the construction and psychometric validation of the “Nursing Competencies Questionnaire on Older People’s Environmental Health” (NCQ‐OPEH). This self‐administered instrument was composed of a knowledge questionnaire, Knowledge Questionnaire on Older People’s Environmental Health (KQ‐OPEH), a skills scale, Skill Scale Older People’s Environmental Health (SS‐OPEH), and an attitude scale, Attitude Scale Older People’s Environmental Health (AS‐OPEH). The process consisted of four phases:

- Phase 1. Creation of the instrument and elaboration of items.
- Phase 2. Content validation through consultation with a panel of experts.
- Phase 3. Pilot test.
- Phase 4. Psychometric validation.

The STROBE checklist for cross‐sectional studies was followed (Supporting File 1).

### 2.2. Phase 1. Creation of the Instrument and Elaboration of Items

First, the construct to be measured (nursing competencies on the effects of climate change and the health of older people) was defined through a review of the available scientific evidence in order to identify the main dimensions that comprised it: knowledge, skills, and attitudes.

To create the knowledge construct, a scoping review was conducted on the effects of climate change on older people’s health [15]. Thus, six domains were identified, composed of several indicators and 34 initial items:

• *Vulnerability of older people* to *the effects of climate change* (11 items).
• *Effect of extreme temperatures* (9 items).
• *Effect of air pollution* (6 items).
• *Synergistic effect of heat and air pollution* (2 items).
• *Effect of extreme weather events on physical and mental health* (4 items).
• *Vector-borne diseases, contaminated water and food* (2 items).

After discussion by the research group, 9 were eliminated, leaving the first version of KQ‐OPEH composed of 25 items (15 true and 10 false). These consisted of statements with mutually exclusive response options (True/False/I don’t know). The “I don’t know” option was included to avoid random responses, increase the reliability of the results, and to be able to subsequently calculate the ignorance index, which reports the degree of ignorance on the subject matter [16].

To construct the skills and attitudes, items, documents, and reports on nursing competencies in environmental health from the following associations were consulted: WHO/PAHO (Pan American Health Organization), CDC (Centers for Disease Control and Prevention), ANA (American Nurses Association) or ANHE (Alliance of Nurses for Healthy Environments), NCEH (National Center for Environmental Health), American Public Health Association, among others. The items on these scales were worded positively to avoid possible confusion among participants since negative wording could make them more difficult to understand or lead to double interpretations [17]. In the first version, four domains were identified for the skills scale (20 items): *Identifying older people at risk from climate-related hazards* (6 items); *general care skills related to climate change* (3 items); *care skills for specific climate-related hazards* (8 items); and *health education on climate change and health risks* (2 items). Similarly, four further areas were established for the scale of attitudes (14 items): *Sustainable healthcare practice* (4 items); *need for professional education on climate change and health* (4 items); *responsibility in caring for older people at risk from climate change* (3 items); and *importance of health education for at-risk populations* (3 items). The items consisted of declarative statements that expressed a statement with which they had to indicate their degree of agreement or disagreement through a bipolar Likert scale where the extremes represented the opposite opinions of the continuum with five possible responses and a neutral midpoint: 1) completely disagree; 2) disagree; 3) neither agree nor disagree; 4) agree; 5) completely agree.

### 2.3. Phase 2. Content Validation

The first version of NCQ‐OPEH was subjected to content validation in order to verify the degree to which the instrument represented the entire construct for which it was developed. The e‐Delphi method was used by consulting a panel of 13 people (9 women and 4 men), experts in climate change and health, social and older people healthcare, questionnaire development, and academics specializing in aging. The panel was consulted through the online platform SurveyMonkey®. They were asked to rate the relevance and clarity of the items according to the following scores on a Likert scale: 1) Not at all relevant/clear; 2) somewhat relevant/clear; 3) quite relevant/clear; and 4) highly relevant/clear. An even number of responses were chosen so that the judges could not evade choosing the central response and would clearly opt for one of the options [18]. They were offered the opportunity to write a comment or alternative wording on each item, and an open field was included at the end of the questionnaire for them to indicate the adequacy of the items or any other comments on the general structure of the instrument. This allowed for a single round of consultation since there were no major discrepancies between the experts, and their suggestions were used to modify the items with a low score on clarity. To obtain agreement between the judges, the Aiken V value was calculated, establishing a threshold of ≥ 0.80 for relevance in the knowledge questionnaire and ≥ 0.90 for the skills and attitudes scales. Those items that did not exceed this value were discarded. Regarding clarity, the threshold was set at ≥ 0.80 for all parts of the instrument, and the items that did not reach this value were rewritten.

### 2.4. Phase 3. Pilot Test

#### 2.4.1. Sample

The NCQ‐OPEH version 2 obtained in the previous phase was applied to a nonprobabilistic convenience sample with similar characteristics to the target population to which the instrument was directed: 35 nurses and 51 nursing university students (86 participants). For data collection, a “snowball” technique was used in nursing professionals and, to guarantee the required students’ sample, the collaboration of teachers from the Nursing Degree was counted on. The link to the instrument hosted on the SurveyMonkey digital platform was distributed using the WhatsApp social network, among professionals, and by QR code in students. Data collection was carried out during September 2023.

#### 2.4.2. Data Analysis

Prior to testing the instrument with the sample, the Inflesz Readability Index was assessed to verify that the level required for its understanding by the sample was appropriate [19].

The correlation of each item with the total score (I–T) was calculated. Additionally, the Difficulty Index (Id) and Discrimination Index (ID) were calculated for the knowledge questionnaire. In each part of the instrument, reliability was assessed using Cronbach’s *α* and McDonald’s *ω*, with values greater than 0.65–0.70 being considered indicators of good internal consistency [20]. In the skills and attitudes scales, the possibility of acquiescence or negative bias (that everyone was totally in favor or against certain items) was also examined. To do this, the response frequency of the different response options in each item should not be > 95% or < 5% [21].

For statistical data analysis, Microsoft Excel and JASP® version 0.17.2.1 programs were used.

### 2.5. Phase 4. Psychometric Validation

#### 2.5.1. Sample

A nonprobabilistic convenience sample of 708 individuals (210 nursing professionals and 498 nursing students) was obtained. Data were collected between January and April 2024. Several strategies were implemented: publication of the link to the instrument through social networks (Instagram, Twitter, and WhatsApp), emails by hospitals to nursing staff, and dissemination of the QR code by nursing degree professors. Data collection via the internet with the SurveyMonkey online platform allowed access to a large number of potential respondents without geographical restriction.

#### 2.5.2. Psychometric Analysis

As in the pilot test, the comprehension validity was first analyzed using the INFLESZ index to check that the instrument still maintained the necessary readability for the sample. In addition, the organization and density of the instrument were evaluated using the PMOSE/IKIRSCH formula, assessing its structure, density, and dependence on factors external to the instrument [22].

Psychometric validation was carried out using Item Response Theory (IRT) in order to obtain a robust, reliable, and precise instrument [21]. The Rasch model was used for KQ‐OPEH, as these are dichotomous items (correct/incorrect; the response option “I don’t know” is scored as incorrect). The Andrich rating scale model (more suitable for Likert scales) was used for SS‐OPEH and AS‐OPEH [23]. The three assumptions on which IRT is based were verified: one‐dimensional construct, through an Exploratory Factor Analysis (EFA); monotonicity or that the probability of responding to an item was a nondecreasing function of the latent phenomenon, through Item Characteristic Curves (ICCs) [24]; and local independence of the items, that is, the responses were not conditioned by each other, observing that the values of the Yen Q3 statistic did not exceed the value of ±0.30 [25].

The latent trait (θ) or ability of the person and the items difficulty (β) were estimated (the average level of difficulty was 0, negative values showed the easiest items and positive values the most difficult ones) [26]. In skill and attitude scales, difficulty index measures the proportion of endorsement, that is, of choice of responses [23]. Both parameters were related in an item map to check that no item was located outside the required level of ability [26].

The data fitting parameters Infit and Outfit were calculated. Although the optimal range of these parameters is between 0.80 and 1.20, values between 0.50 and 1.50 were considered acceptable. In any case, it was taken into account that the values were not greater than 2 because they would degrade the measurement, nor less than 0.50, because they would artificially inflate it [23, 26].

Construct validity was also assessed using EFA. The data were previously checked for factorability using the Kaiser–Meyer–Olkin (KMO) test (with a value of > 0.60) and Bartlett’s test of sphericity, which was significant (*p* < 0.05). Principal component analysis was realized using parallel analysis with the simulation performed by the free Monte Carlo PCA software.

To measure the quality of the instruments, the separation index and reliability were studied for both items and people. Reliability values higher than 0.70 and 2 in the case of the separation index were sought [27].

Additionally, the internal consistency value was provided for each of the parts of the instrument. Specifically, Cronbach’s *α* and McDonald’s *ω* values were reported, with values above 0.65 being considered acceptable [20].

All parameters and statistics discussed above were calculated using the jMetrik® and JASP® programs.

The database corresponding to the psychometric validation of the instrument is available in the repository RUJA through the handle: https://hdl.handle.net/10953/6471.

Finally, a proposal was made to categorize the instrument scores and to be able to classify the results into five levels based on the percentage of maximum score:

• KQ‐OPEH: Excellent knowledge: > 90%; very good knowledge: 89%–80%; good knowledge: 79%–60%; insufficient knowledge: 59%–40%; poor knowledge: < 40%.
• SS‐OPEH and AS‐OPEH: Excellent skills/attitudes: > 90%; very good skills/attitudes: 89%–80%; good skills/attitudes: 79%–70%; insufficient skills/attitudes: 69%–50%; poor skills/attitudes: < 50%.

### 2.6. Ethical Considerations

The study was approved by the Andalusian Biomedical Research Ethics Committee (PEIBA) (code: Tesis‐CCECCSM‐2019/0902‐N‐21 dated: 10/28/2021).

The objective, nature, and purpose of the study were transmitted to the participants at the time of accessing the link to the instrument. They were informed that by completing the questionnaire and the scales they were giving their consent for completely voluntary participation in the research and that the information would be used exclusively for scientific purposes, ensuring the participants’ anonymity and the confidentiality of the data, which were not accessible to people outside the study.

## 3. Results

### 3.1. Content Validation

After analyzing the index of agreement between the judges (Supporting File 2), five knowledge items, five skills items, and two attitude items were eliminated because they did not reach the minimum value established for relevance. Additionally, the wording of 10, 4, and 1 items, respectively, was modified, either because they did not exceed the value established for clarity or because the comments provided by the experts significantly improved the understanding of the items.

At the end of the content validation phase, Version 2 of NCQ‐OPEH instrument was obtained, as follows: 20 items in KQ‐OPEH (13 true and 7 false), 15 in SS‐OPEH, and 12 in AS‐OPEH.

### 3.2. Pilot Test

The INFLESZ index obtained a “somewhat difficult” readability score, which corresponded to a university‐level comprehension level, suitable for the target population.

The sample consisted of 59.3% students and 40.7% nursing professionals. Furthermore, 79.07% of the population were women.

#### 3.2.1. Item Statistical Analysis

The KQ‐OPEH knowledge questionnaire consisted of nine very easy items (45%), three easy items (20%), two medium‐difficult items (10%), and three very difficult items (15%). Regarding the Discrimination Index, six items (30%) had very poor or terrible discriminatory capacity, five had regular discrimination (25%), and nine had good or excellent discrimination capacity (45%). No item had an I–T score greater than 0.80, which meant that there were no repetitive items (Supporting File 3). Regarding reliability, the questionnaire obtained Cronbach’s *α* value of 0.75 (95% CI 0.67–0.82) and McDonald’s *ω* of 0.76 (95% CI 0.68–0.83), demonstrating good internal consistency. After this analysis, it was decided to eliminate five items because they were too easy, had poor or no discriminatory capacity, and/or their classification with the total score was below the set critical value of 0.30.

The SS‐OPEH skills scale and the AS‐OPEH attitudes scale items all correlated positively with the total I–T score, with values above 0.30. No statement presented correlation values above 0.8 (Supporting File 3). The scales obtained reliability values ≥ 0.90 (Cronbach’s *α* = 0.90 (95% CI, 0.87–0.93) and 0.91 (95% CI, 0.87–0.93); McDonald’s *ω* = 0.90 (95% CI, 0.87–0.93) and 0.91 (95% CI, 0.88–0.94)) showing excellent internal consistency. It was decided not to eliminate any item from these two scales since all of them showed adequate correlation values with the total score, as well as excellent internal consistency.

At the end of the pilot phase, Version 3 of NCQ‐OPEH instrument consisted of KQ‐OPEH with 15 items (11 true and 4 false); SS‐OPEH, 15 items, and AS‐OPEH, 12 items.

### 3.3. Psychometric Validation

Both the INFLESZ index and the PMOSE/IKIRSCH formula showed an appropriate level of complexity for its application in nurses and nursing students.

Sample sociodemographic characteristics are shown in Table 1.

**TABLE 1:** Participants’ sociodemographic characteristics.

Characteristic	Value
Nurses, *n* (%)	210 (29.66)
Nursing students, *n* (%)	498 (70.34)
Mean age (years)	29.13 (SD* = 12.66*)
Nurses’ mean age (years)	43.81 (SD* = 10.90*)
Mean age (years)	22.94 (SD* = 7.02*)
Gender, *n* (%):	
Man	128 (18.08)
Woman	573 (80.93)
Transsexual	1 (0.14)
Nonbinary	4 (0.57)
Other	2 (0.28)
Training in climate change and health, *n* (%):	
No	473 (66.81)
Yes	235 (33.19)

**Nurses**	

Professional experience (years)	18.68 (*SD = 10.77*)
Qualification, *n* (%)	
U.D.N	134 (63.81)
Degree in Nursing	76 (36.19)
Workplace, *n* (%):	
Health centers	54 (25.71)
Nursing Homes	8 (3.81)
Hospital Care	115 (54.76)
Other	33 (15.72)

**Nursing students**	

Degree access qualification, *n* (%):	
High school	239 (47.99)
Vocational training	215 (43.17)
Previous university qualification	14 (2.81)
Other	30 (6.03)
Highest course enrolled, *n* (%):	
First	162 (32.53)
Second	184 (36.95)
Third	133 (26.71)
Fourth	19 (3.81)
Assistance practices, *n* (%):	
No	338 (67.87)
Yes	32 (32.13)

*Note:* U.D.N: University Diploma in Nursing (Former degree qualification).

#### 3.3.1. Psychometric Analysis

**FIGURE 1:**
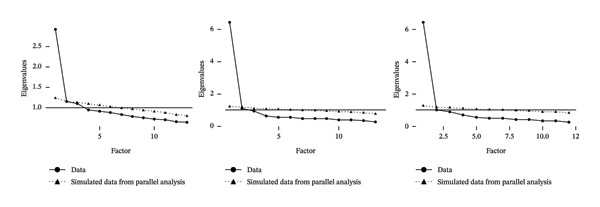
NCQ‐OPEH scree plots. Scree plots comparing the AFE eigenvalues with those obtained by random parallel simulation.

It was necessary to remove two knowledge items and two skill items to achieve the unidimensionality of the construct using AFE. The EFA was repeated, showing that KQ‐OPEH, SS‐OPEH, and AS‐OPEH had only one dimension each, as shown in Figure 1.

Yen’s Q3 statistic correlation values showed the local independence of the items (Supporting File 4). Only one attitude item obtained a correlation value slightly higher than 0.30 (0.35 and 0.33), so it was a candidate for elimination.

As for monotony, knowledge item characteristic curves represented increasing functions, and in the skills and attitudes scales, the set of curves corresponding to the different response categories shows how they intersect in an orderly manner (Supporting File 5). Thus, the third assumption was demonstrated.

Using the Rasch model, two items that showed high outfit values had to be discarded. After repeating the analysis, the mean for the infit statistic was 1.00 (0.84–1.23) and that of outfit, 1.05 (0.77–1.58), within the acceptable range. The difficulty ranged between −2.23 for the easiest item and 2.85 for the most difficult (Table 2). The separation index for the items was 14.24 and 1.10 for people. A reliability statistic value of 0.99 was obtained for the items, while for people it was 0.54.

**TABLE 2:** KQ‐OPEH Rasch model parameters.

Item	Difficulty (SE)	Infit	Outfit
People over 65 years of age are part of the population most vulnerable to the climate change effects.	−1.90 (0.12)	1.03	1.10
Socioeconomic factors affect older people’s ability to adapt to climate change risk.	−2.23 (0.13)	1.01	1.01
Climate change consequences affect older women’s health more than same age men.	1.64 (0.09)	0.96	1.09
Polypharmacy increases the likelihood of premature death in older people at risk due to climate change.	−0.71 (0.09)	0.94	0.84
Extreme heat is the climate change effect that most affects the older people’s health.	0.28 (0.09)	1.05	1.14
Intense heat causes adverse health effects on older people more acutely than extreme cold.	1.45 (0.09)	1.23	1.43
Dementia hospitalization risk increases when there is prolonged exposure to low temperatures.	2.85 (0.12)	1.16	1.58
Chronic exposure to air pollution related to heavy traffic is a risk factor for the onset of cognitive impairment in older people.	−0.34 (0.09)	0.90	0.80
There is a relationship between prolonged exposure to polluted air and anxiety high levels in older people.	−0.04 (0.09)	0.84	0.77
The population over 65 years of age is especially vulnerable to contracting gastrointestinal infections due to contaminated water.	−0.74 (0.09)	0.98	0.90
There is a statistical relationship between experiencing an extreme weather event and the appearance of post‐traumatic stress in older people.	−0.27 (009)	0.93	0.87

*Note:* Infit: weighted mean square fit; outfit: unweighted mean square fit. Items are a simple translation of the Spanish original.

Abbreviation: SE, standard error.

For the SS‐OPEH skills scale, the infit mean value was 1.00 (ranging from 0.77 to 1.19) and 1.00 for outfit (0.80–1.23). Difficulty ranged from −0.92 for the most popular item to 1.02 for the most unpopular item (Table 3). The separation index was strong, both between items (10.49) and between individuals (3.07). Reliability for the set of items and for individuals was excellent (0.99 and 0.90, respectively).

**TABLE 3:** SS‐OPEH parameters of Andrich’s rating scale model.

Item	Difficulty (SE)	Infit	Outfit
I am able to recognize what factors of climate change that pose a risk to the health of older people.	−0.64 (0.06)	1.16	1.16
I am able to identify those older people who are most exposed to the climate change effects.	−0.62 (0.06)	1.03	1.00
I am able to develop care plans focusing on the specific climate risks to which older people are exposed.	0.41 (0.05)	1.01	1.07
I am able to identify the climate change effects that may affect the mental health of older people.	0.17 (0.05)	0.99	1.03
I am able to recognize in older people the side effects of medication that aggravate conditions attributable to climate change.	1.02 (0.05)	0.96	0.98
I am able to identify the risks posed by high temperatures to the cognitive status of older people.	−0.68 (0.06)	1.05	1.03
I am able to assess the housing conditions that pose a risk to older people in case of extreme temperatures.	−0.92 (0.06)	1.09	1.05
I can distinguish the effects of different air pollutants on the respiratory health of older people.	0.56 (0.05)	1.03	1.03
I am able to query environmental pollutant level to alert older people with chronic respiratory diseases.	0.76 (0.05)	1.19	1.23
I am able to assess the environmental threats of climate change that aggravate the older people’s cardiovascular health.	0.34 (0.05)	0.77	0.80
I am able to carry out the necessary actions to combat environmental risks in a nursing home of older people.	0.32 (0.05)	0.78	0.80
I am able to carry out health education for older people in order to reduce their health risks.	−0.49 (0.06)	1.02	0.99
I am able to conduct health education for older people on the effects of climate change on their health.	−0.23 (0.06)	0.90	0.87

*Note:* Infit: weighted mean square fit; outfit: unweighted mean square fit. Items are a simple translation of the Spanish original.

Abbreviation: SE, standard error.

For the AS‐OPEH attitude scale, the Andrich model’s parameters showed one item with infit and outfit values above the recommended (2.48 and 2.80, respectively). That item was the same one that had correlation values greater than 0.30, so it was discarded, and the analysis was repeated. After this, the infit mean was 1.00 (from 0.61 to 1.18) and the outfit mean was 1.01 (0.57–1.31), both in the range of optimal values. The difficulty values ranged from −0.52 to 0.23 (Table 4). The separation index was > 2 for both items and persons (4.41 and 2.70, respectively). Reliability was excellent for items (0.95) and very good for persons (0.88).

**TABLE 4:** Andrich model parameters for AS‐OPEH.

Item	Difficulty (SE)	Infit	Outfit
Health professionals have the responsibility to provide sustainable care to older people in order to contribute to the fight against climate change.	−0.29 (0.08)	1.18	1.31
Health professionals can take different actions to prevent the effects of climate change from negatively impacting the health of older people.	−0.52 (0.08)	1.03	1.08
Health professionals require specific knowledge about the health problems caused by extreme temperatures in older people.	−0.10 (0.07)	0.80	0.85
Health professionals need specific training on climate change and older people’s health.	−0.09 (0.07)	0.73	0.78
Health professionals need to be informed about the potential environmental hazards to which older people are exposed.	−0.45 (0.08)	0.63	0.66
It is important that health professionals are aware of climate change effect consequences on the health of older people.	−0.38 (0.08)	0.61	0.57
Health professionals are uniquely placed to help older people at climate risk through adaptation plans.	0.02 (0.07)	1.10	1.14
Health professionals have the responsibility to address climate change impacts on the health of the older population.	0.23 (0.07)	0.91	0.94
Interventions by health professionals can reduce the health‐related effects of climate change on older people’s health.	−0.02 (0.07)	1.04	1.08
Health professionals should educate older people about creating healthy environments to prevent climate change–related diseases.	−0.09 (0.07)	0.69	0.71
Health professionals have the responsibility to provide sustainable care to older people in order to contribute to the fight against climate change.	−0.15 (0.07)	0.73	0.74

*Note:* Infit: weighted mean square fit; outfit: unweighted mean square fit. Items are a simple translation of the Spanish original.

Abbreviation: SE, standard error.

Threshold analysis on the Likert scale showed that they ranged from a minimum of −2.56 and −2.41 to a maximum of 3.41 and 3.24 in SS‐OPEH and AS‐OPEH, respectively. These thresholds increased progressively and without regression in the two scales. The fit of the response categories was good, with all infit and outfit values within the optimal range.

**FIGURE 2:**
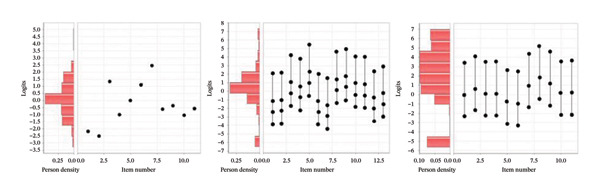
NCQ‐OPEH item maps. In the item maps, the level of skill or latent trait (θ) of the sample (ordinate axis) is related to the difficulty (β) of the items (abscissa axis).

The item map shows that all items were located within the range of the participants’ ability level, i.e., the difficulty index was appropriate for measuring the latent trait of the sample (Figure 2).

Finally, internal consistency of each part of the instrument was calculated, obtaining adequate reliability values for KQ‐OPEH and excellent reliability values for SS‐OPEH and AS‐OPEH in the two calculated statistics (Table 5).

**TABLE 5:** NCQ‐OPEH reliability values.

	KQ‐OPEH		SS‐OPEH		AS‐OPEH	
	Value	95% CI	Value	95% C	Value	95% CI
Cronbach’s *α*	0.68	0.64–0.71	0.91	0.90–0.92	0.93	0.92–0.94
McDonald’s *ω*	0.68	0.65–0.72	0.91	0.91–0.92	0.93	0.92–0.94

The final NCQ‐OPEH instrument (available at https://hdl.handle.net/10953/7389) was composed of:

- KQ‐OPEH: 11 items (9 true and 2 false).
- SS‐OPEH: 13 items.
- AS‐OPEH: 11 items.

## 4. Discussion

In the present study, a new tool to measure nursing competencies on the climate change effects on older people’s health was developed and psychometrically validated. This instrument, composed of a knowledge questionnaire KQ‐OPEH, a skills scale SS‐OPEH, and an attitude scale AS‐OPEH, was subjected to content validation by a panel of experts, a pilot test, and a psychometric validation using IRT, widely used for questionnaire validation studies [14, 28, 29]. The instrument showed a strong separation between items, although with some restrictions for people, which may be due to the imbalance between the number of nursing professionals and nursing students in the sample. Therefore, future research will aim to increase the population of nurses while further increasing the sample of the two groups, both nationally and internationally. In terms of reliability, the skills and attitudes scales obtained excellent internal consistency, 0.91 and 0.93, respectively. In the knowledge questionnaire, it was acceptable (0.68), although somewhat lower than in the pilot test (0.75), probably affected by a smaller number of items [30]. We found no similar tool with which to assess criterion validity.

The descriptive analysis showed that, in the sample studied, slightly more than half of the participants obtained scores corresponding to an insufficient to poor level of knowledge (53.69%) and skills (55.06%). However, the vast majority of participants showed good to excellent attitudes (91.18%). Research on knowledge of climate change and its impact on health concluded that the majority of health professionals [31, 32] and students [33] had a moderate knowledge of climate change. However, other studies concluded that the majority of professionals [34] and nursing students had insufficient [35, 36] or moderate knowledge [33]. A study on nursing competencies in children’s environmental health found similar results to ours, with just over 50% of students having insufficient or deficient skills [36]. The highest skill reported was, as in our case, related to identifying air pollutants causing respiratory diseases in these vulnerable populations. In the research on environmental attitudes, most of the participants reported a high level of concern about the issue and considered it necessary to acquire knowledge about it [31]. However, very few professionals made behavioral changes at home or at work. Research focusing on nursing students revealed that they had moderate proenvironmental attitudes and very positive attitudes toward sustainable healthcare [33]. Despite this, most did not engage in behaviors to mitigate environmental risks, although they agreed on the need to include climate change education in the nursing curriculum [35, 36]. Therefore, the people studied showed good attitudes, but less knowledge and skills, especially in the student group. They seemed to be aware that climate change has a serious impact on older people’s health; however, they lacked knowledge about the particularities of the effects on this population group and the skills needed to provide adequate care for them.

The NCQ‐OPEH is a comprehensive tool to identify gaps in the current education and training of nurses in this specific area. It also makes it possible to assess the effect of educational and learning interventions on environmental nursing competencies, as several previous studies evaluating the effectiveness of different types of training sessions, aimed at nurses or students, have done [37, 38]. By measuring knowledge, skills, and attitudes before and after participating in training programs, the effectiveness of such programs can be determined and the necessary adjustments made to optimize their performance. This continuous evaluation would ensure that nurses remain up to date with the latest scientific advances and best practices in relation to climate change and the health of older people. Applied to nursing students, NCQ‐OPEH would provide specific information about the content that should be incorporated into the academic curriculum during their university education for the acquisition of these environmental competencies.

### 4.1. Strengths and Limitations of the Study

Our study presents some strengths to be taken into account. First, the fact that it is designed to be self‐administered ensures the absence of researcher and observer bias, all the more so because of the anonymity of the participants. Second, the wide variety of strategies employed in the recruitment of the sample makes it easier to make the sample as representative as possible. This is also reinforced by the use of social networks and online platforms, as this meant that there were no geographical restrictions during the recruitment of both experts and nurses. Third, the application of IRT is more rigorous than is usually used in similar work on the development of scales in nursing and in the evaluation of climate and health. In addition, the multiphase development (content validation via e‐Delphi, pilot testing, and psychometric validation) follows established best practices for instrument design. Fourth, the validation process was performed independently for each component of the instrument. It makes it possible to apply the complete instrument if the aim is to measure the set of environmental competencies, or each of its parts separately, if the aim is to assess any of the competencies (knowledge, skills, or attitudes) in a specific way. Additionally, as the study was carried out with both samples, it can be used both with nurses, from any context and level of healthcare, and with university nursing students. The instrument created in this study fills a clear gap by focusing specifically on nursing competencies related to the impacts of climate change on older adults, a highly vulnerable population with growing demographic relevance.

On the other hand, the type of nonprobability sampling used may have resulted in some parameters and indices showing slightly lower values for individuals. Furthermore, as this is a novel instrument, there is no gold standard with which to examine its convergent validity. Therefore, in future research, we will try to use a more appropriate sampling technique and increase the number of nurses.

## 5. Conclusions

The NCQ‐OPEH is the first instrument capable of reliably measuring competencies (knowledge, skills, and attitudes) regarding the effects of climate change on the health of older people, both in nurses and in nursing students.

In general, nurses and nursing students had insufficient knowledge and skills. However, they had excellent attitudes toward the important role of nursing in providing care to the older population at risk from climate change.

## Author Contributions

All authors made important contributions to the present study, as shown below: Eva M. Montoro‐Ramírez: conceptualization, investigation, data curation, project administration, funding acquisition, resources, supervision, formal analysis, methodology, software, visualization, writing–original draft, and writing–review and editing. Laura Parra‐Anguita: conceptualization, data curation, investigation, resources, supervision, formal analysis, validation, methodology, software, visualization, writing–original draft and writing–review and editing. Isabel M. López‐Medina: conceptualization, data curation, investigation, funding acquisition, resources, supervision, methodology, software, validation, visualization, writing–original draft, and writing–review and editing. Carmen Álvarez‐Nieto: conceptualization, investigation, visualization, and writing–review and editing.

## Funding

Eva M. Montoro‐Ramírez reports a grant from the Ministry of Science, Innovation and Universities of the Government of Spain [grant number FPU 19/01871].

## Disclosure

All authors have approved the version to be published, agreed to be responsible for the information presented in the work, and have guaranteed that any problems of accuracy or completeness are properly resolved.

## Conflicts of Interest

The authors declare no conflicts of interest.

## Supporting Information

Additional supporting information can be found online in the Supporting Information section.

## Supporting information


**Supporting Information 1** Supporting File 1. STROBE Statement—Checklist of items that should be included in reports of cross‐sectional studies.

**Supporting Information 2** Supporting File 2. Agreement Index among experts in content validation.

**Supporting Information 3** Supporting File 3. Items statistical analysis in the Pilot test.

**Supporting Information 4** Supporting file 4. Correlation Yen’s Q3 Statistic.

**Supporting Information 5** Supporting file 5. NQC‐OPEH’s Items Characteristic Curves.

## Data Availability

The data that support the findings of this study are openly available in the Institutional Repository of Scientific Output of the University of Jaén (RUJA) at https://hdl.handle.net/10953/6471. The instrument Nursing Competencies Questionnaire on Older People’s Environmental Health (NCQ‐OPEH) is openly available in the Institutional Repository of Scientific Output of the University of Jaén (RUJA) and can be consulted at https://hdl.handle.net/10953/7389.

## References

[1] Sánchez González D. and Chávez Alvarado R. , Envejecimiento De La Población Y Cambio Climático, 2019, Comares, S.L, Granada.

[2] Ayalon L. , Keating N. , Pillemer K. , and Rabheru K. , Climate Change and Mental Health of Older Persons: A Human Rights Imperative, American Journal of Geriatric Psychiatry. (2021) 29, no. 10, 1038–1040, 10.1016/j.jagp.2021.06.015. 34294541

[3] United Nations Department of Economic and Social Affairs Population Division , World Population Prospects 2022: Summary of Results, UN. (2022) https://www.un.org/development/desa/pd/content/World-Population-Prospects-2022.

[4] Sayre L. , Rhazi N. , Carpenter H. , and Hughes N. L. , Climate Change and Human Health: The Role of Nurses in Confronting the Issue, Nursing Administration Quarterly. (2010) 34, no. 4, 334–342, 10.1097/NAQ.0b013e3181f60df9. 20838179

[5] Organización Panamericana de la Salud , Cambio Climático Para Profesionales De La Salud: Un Libro De Bolsillo, 2020, OPS, Washington, DC, https://iris.paho.org/handle/10665.2/52950.

[6] López-Medina I. M. , Álvarez-Nieto C. , Grose J. et al., Competencies on Environmental Health and Pedagogical Approaches in the Nursing Curriculum: A Systematic Review of the Literature, Nurse Education in Practice. (2019) 37, 1–8, 10.1016/j.nepr.2019.04.004. 31002889

[7] Álvarez-García C. , López-Medina I. M. , Sanz-Martos S. , and Álvarez-Nieto C. , Salud Planetaria: Educación Para Una Atención Sanitaria Sostenible, Educación Médica. (2021) 22, no. 6, 352–357, 10.1016/j.edumed.2021.08.001.

[8] Guzman C. A. , Potter T. , Aguirre A. et al., The Planetary Health Education Framework, Planetary Health Alliance. (2021) 5, no. 5, 253–255, 10.5822/phef2021.

[9] Portela dos Santos O. , Perruchoud É. , Pereira F. , Alves P. , and Verloo H. , Measuring Nurses’ Knowledge and Awareness of Climate Change and Climate-Associated Diseases: Systematic Review of Existing Instruments, Nurs Rep. (2024) 14, no. 4, 2850–2876, 10.3390/nursrep14040209. 39449446 PMC11503396

[10] Roberge M. , Diallo T. , Bérubé A. , Audate P. P. , and Leblanc N. , Climate Change Integration in Nursing Academic Curricula and Continuing Education: A Scoping Review, Canadian Journal of Nursing Research. (2025) 57, no. 3, 406–428, 10.1177/08445621251341646. PMC12344213 40528665

[11] Schenk E. , Butterfield P. , Postma J. , Barbosa-Leiker C. , and Corbett C. , Creating the Nurses’ Environmental Awareness Tool (NEAT), Workplace Health & Safety. (2015) 63, no. 9, 381–391, 10.1177/2165079915592071. 26215976

[12] Schenk E. C. , Cook C. , Demorest S. , and Burduli E. , CHANT: Climate, Health, and Nursing Tool: Item Development and Exploratory Factor Analysis, Annual Review of Nursing Research. (2020) 38, no. 1, 97–112, 10.1891/0739-6686.38.97. 32102957

[13] Vrotsou K. , Subiza-Pérez M. , Lertxundi A. et al., Environmental Health Knowledge of Healthcare Professionals: Instrument Development and Validation Using the Rasch Model, Environmental Research. (2023) 235, 10.1016/j.envres.2023.116582. 37454800

[14] Álvarez-García C. , Álvarez-Nieto C. , Pancorbo-Hidalgo P. L. , Sanz-Martos S. , and López-Medina I. M. , Student Nurses′ Knowledge and Skills of Children′s Environmental Health: Instrument Development and Psychometric Analysis Using Item Response Theory, Nurse Education Today. (2018) 69, 113–119, 10.1016/j.nedt.2018.07.008. 30036709

[15] Montoro-Ramírez E. M. , Parra-Anguita L. , Álvarez-Nieto C. , Parra G. , and López-Medina I. M. , Climate Change Effects in Older People’s Health: A Scoping Review, Journal of Advanced Nursing. (2024) 00, no. 12, 1–14, 10.1111/jan.16270. PMC12623704 38895960

[16] Muñoz T. G. , El Cuestionario Como Instrumento De investigación/Evaluación, Centro Universitario Santa Ana. (2003) 1, no. 1, 1–47, http://www.etpcba.com.ar/documentos/sitios/evaluacion_intitucional/8_el_cuestionario.pdf.

[17] Delgado S. C. , Marín B. M. , Sánchez J. , and Ramos L. , Métodos De Investigación Y Análisis De Datos En Ciencias Sociales Y De La Salud, 2011, Pirámide, España.

[18] Romero-Collado A. , Elementos Esenciales Para Elaborar Un Estudio Con El Método (E), Delphi. Enferm. intensiva.(2021) 32, no. 2, 100–104, 10.1016/j.enfi.2020.09.001. PMC7525331 33008751

[19] Barrio-Cantalejo I. M. , Simón-Lorda P. , Melguizo M. , Escalona I. , Marijuán M. I. , and Hernando P. , Validación De La Escala INFLESZ Para Evaluar La Legibilidad De Los Textosdirigidos a Pacientes, Anales Sis San Navarra. (2008) 31, no. 2, 135–152, https://scielo.isciii.es/scielo.php?script=sci_arttext%26pid=S1137_66272008000300004%26lng=es, Available at 10.4321/s1137-66272008000300004. 18953362

[20] Katz M. H. , Multivariable Analysis: A Practical Guide for Clinicians and Public Health Researchers, 2011, 3rd edition, Cambridge University Press, 10.1017/CBO9780511974175.

[21] Kyriazos T. A. and Stalikas A. , Applied Psychometrics: The Steps of Scale Development and Standardization Process, Psychology. (2018) 9, no. 11, 2531–2560, 10.4236/psych.2018.911145.

[22] Mosenthal P. B. and Kirsch I. S. , A New Measure for Assessing Document Complexity: the PMOSE/IKIRSCH Document Readability Formula, Journal of Adolescent & Adult Literacy. (1998) 41, no. 8, 638–657.

[23] Meyer J. P. , Applied Measurement with Jmetrik, 2014, Routledge, New York, 10.4324/9780203115190.

[24] Matas-Terrón A. , Introducción Al Análisis De La Teoría De Respuesta Al Ítem. Aidesoc, 2010, http://hdl.handle.net/10630/4711.

[25] Lalinde J. D. H. , Castro F. E. , Rodríguez J. E. et al., Sobre El Uso Adecuado Del Coeficiente De Correlación De Pearson: Definición, Propiedades Y Suposiciones, Archivos Venezolanos de Farmacología y Terapéutica. (2018) 37, no. 5, 587–595.

[26] Prieto Adánez G. A. and Delgado González A. R. , Análisis De Un Test Mediante El Modelo De Rasch, Psicothema. (2003) 15, no. 1, 94–100.

[27] Streiner D. L. , Norman G. R. , and Cairney J. , Health Measurement Scales: A Practical Guide to Their Development and Use, 2024, Oxford University Press, 5th edn, Available at 10.1093/med/9780199685219.001.0001.

[28] Liu T. , Ho A. D. , Hsu Y. , and Hsu C. , Validation of the EQ-5D in Taiwan Using Item Response Theory, BMC Public Health. (2021) 21, no. 1, 10.1186/s12889-021-12334-y. PMC8684691 34923963

[29] López Gómez M. A. L. , Gundersen D. A. , Boden L. I. et al., Validation of the Workplace Integrated Safety and Health (WISH) Assessment in a Sample of Nursing Homes Using Item Response Theory (IRT) Methods, BMJ Open. (2021) 11, no. 6, 10.1136/bmjopen-2020-045656. PMC8215260 34145013

[30] Muñiz J. , Introducción a La Psicometría, 2018, Difusora Larousse- Ediciones Pirámide, Madrid.

[31] Xiao J. , Fan W. , Deng Y. , Li S. , and Yan P. , Nurses′ Knowledge and Attitudes Regarding Potential Impacts of Climate Change on Public Health in Central of China, International Journal of Nursing Science. (2016) 3, no. 2, 158–161, 10.1016/j.ijnss.2016.04.002.

[32] Luque-Alcaraz O. M. , Aparicio-Martínez P. , Gomera A. , and Vaquero-Abellán M. , The Environmental Awareness of Nurses as Environmentally Sustainable Health Care Leaders: a Mixed Method Analysis, BMC Nursing. (2024) 23, no. 1, 10.1186/s12912-024-01895-z. PMC10988952 38570845

[33] Felicilda-Reynaldo R. F. D. , Cruz J. P. , Alshammari F. et al., Knowledge of and Attitudes Toward Climate Change and its Effects on Health Among Nursing Students: A Multi-Arab Country Study, Nursing Forum. (2018) 53, no. 2, 179–189, 10.1111/nuf.12240. 28950412

[34] Buriro N. A. , Mureed S. , Kumar R. , Ahmed F. , Hussain K. , and Fatima A. , Nurses’ Perception, Knowledge and Information Sources on Climate Change and Health at Dow University Hospital Karachi, JLUMHS. (2018) 17, no. 04, 265–271, 10.22442/jlumhs.181740590.

[35] Ryan E. C. , Dubrow R. , and Sherman J. D. , Medical, Nursing, and Physician Assistant Student Knowledge and Attitudes Toward Climate Change, Pollution, and Resource Conservation in Health Care, BMC Medical Education. (2020) 20, 1–14, 10.1186/s12909-020-02099-0. PMC7310528 32576175

[36] Álvarez-García C. , Álvarez-Nieto C. , Sanz-Martos S. et al., Undergraduate Nursing Students′ Attitudes, Knowledge, and Skills Related to Children’s Environmental Health, Journal of Nursing Education. (2019) 58, no. 7, 401–408, 10.3928/01484834-20190614-04. 31242309

[37] Richardson J. , Heidenreich T. , Álvarez-Nieto C. et al., Including Sustainability Issues in Nurse Education: A Comparative Study of First Year Student Nurses’ Attitudes in Four European Countries, Nurse Education Today. (2016) 37, 15–20, 10.1016/j.nedt.2015.11.005. 26646207

[38] Álvarez-García C. , Álvarez-Nieto C. , Kelsey J. , Carter R. , Sanz-Martos S. , and López-Medina I. M. , Effectiveness of the e-NurSus Children Intervention in the Training of Nursing Students, International Journal of Environmental Research and Public Health. (2019) 16, no. 21, 10.3390/ijerph16214288. PMC6862581 31694191

