# Supplementary material for: Creation and Psychometric Validation of “Nursing Competencies Questionnaire on Older People’s Environmental Health (NCQ‐OPEH)” in Nurses and Nursing Students
Source: Nurs Res Pract. 2026 Jul 24;2026:1783950. doi: 10.1155/nrp/1783950 (PMC13397473; doi:10.1155/nrp/1783950)
Supplement: Supplementary file 2 — Supporting Information 2 Supporting File 2. Agreement Index among experts in content validation. [file NRP-2026-1783950-s002.docx]

**Supplementary File 2.** Agreement Index among experts in content validation.

***KQ-OPEH:***

| Item | Relevance | | | Clarity | |
| --- | --- | --- | --- | --- | --- |
|  | V_Aiken | | 95% CI | V_Aiken | 95% CI |
| C1 | 0.85 | 0.70-0.93 | | 0.90 | 0.76-0.96 |
| C2 | 0.90 | 0.76-0.96 | | 0.90 | 0.76-0.96 |
| C3 | 0.90 | 0.76-0.96 | | 0.85 | 0.70-0.93 |
| C4 | 0.95 | 0.83-0.99 | | 0.92 | 0.80-0.97 |
| C5 | 0.92 | 0.80-0.97 | | 0.92 | 0.80-0.97 |
| C6 | 0.92 | 0.80-0.97 | | 0.92 | 0.80-0.97 |
| C7 | 0.85 | 0.70-0.93 | | 0.82 | 0.80-0.97 |
| C8 | 0.82 | 0.67-0.91 | | 0.92 | 0.67-0.91 |
| C9 | 0.97 | 0.87-1 | | 0.95 | 0.80-0.97 |
| C10 | 0.90 | 0.76-0.96 | | 0.90 | 0.83-0.89 |
| C11 | 0.95 | 0.83-0.99 | | 0.95 | 0.76-0.96 |
| C12 | **0.74^*^** | 0.59-0.85 | | 0.90 | 0.83-0.99 |
| C13 | 0.87 | 0.73-0.94 | | 0.87 | 0.76-0.96 |
| C14 | 0.87 | 0.73-0.94 | | 0.90 | 0.73-0.94 |
| C15 | **0.79^*^** | 0.64-0.89 | | 0.85 | 0.76-0.96 |
| C16 | 0.82 | 0.67-0.91 | | 0.85 | 0.70-0.93 |
| C17 | 0.95 | 0.83-0.99 | | 0.90 | 0.70-0.93 |
| C18 | **0.72^*^** | 0.56-0.83 | | 0.87 | 0.76-0.96 |
| C19 | 0.85 | 0.70-0.93 | | 0.87 | 0.73-0.94 |
| C20 | 0.82 | 0.67-0.91 | | 0.90 | 0.76-0.96 |
| C21 | **0.64^*^** | 0.48-0.77 | | **0.79^**^** | 0.64-0.89 |
| C22 | 0.82 | 0.67-0.91 | | 0.82 | 0.67-0.91 |
| C23 | **0.79^*^** | 0.64-0.89 | | **0.79^**^** | 0.64-0.89 |
| C24 | 0.82 | 0.67-0.91 | | 0.85 | 0.70-0.93 |
| C25 | 0.92 | 0.80-0.97 | | 0.97 | 0.87-1 |

**Bold*:** less than the Aiken V value limit set in Relevance (≥0.80)

**Bold**:** less than the Aiken V value limit set in Clarity (≥0.80)

***SS-OPEH:***

| Item | Relevance | | | Clarity | |
| --- | --- | --- | --- | --- | --- |
|  | V_Aiken | | 95% CI | V_Aiken | 95% CI |
| H1 | 0.95 | 0.83-0.99 | | 0.90 | 0.76-0.96 |
| H2 | **0.82^*^** | 0.67-0.91 | | **0.79^**^** | 0.64-0.89 |
| H3 | 0.97 | 0.87-1 | | 0.90 | 0.76-0.96 |
| H4 | 0.92 | 0.87-1 | | 0.92 | 0.80-0.97 |
| H5 | 0.92 | 0.80-0.97 | | 0.92 | 0.80-0.97 |
| H6 | 0.95 | 0.83-0.99 | | **0.79^**^** | 0.64-0.89 |
| H7 | **0.87^*^** | 0.73-0.94 | | **0.79^**^** | 0.64-0.89 |
| H8 | 0.90 | 0.76-0.96 | | **0.74^**^** | 0.59-0.85 |
| H9 | 0.90 | 0.76-0.96 | | **0.74^**^** | 0.59-0.85 |
| H10 | 0.97 | 0.87-1 | | 0.97 | 0.87-1 |
| H11 | 0.97 | 0.87-1 | | 0.97 | 0.87-1 |
| H12 | 0.90 | 0.76-0.96 | | 0.95 | 0.83-0.99 |
| H13 | 0.90 | 0.76-0.96 | | **0.72^**^** | 0.56-0.83 |
| H14 | 0.92 | 0.80-0.97 | | 0.90 | 0.76-0.96 |
| H15 | **0.79^*^** | 0.64-0.89 | | **0.72^**^** | 0.56-0.83 |
| H16 | **0.87*** | 0.73-0.94 | | **0.74^**^** | 0.59-0.85 |
| H17 | 0.92 | 0.80-0.97 | | 0.85 | 0.70-0.93 |
| H18 | 0.95 | 0.83-0.99 | | 0.90 | 0.76-0.96 |
| H19 | 0.95 | 0.83-0.99 | | 0.90 | 0.76-0.96 |
| H20 | **0.69*** | 0.54-0.81 | | 0.85 | 0.70-0.93 |

**Bold*:** less than the Aiken V value limit set in Relevance (≥0.90)

**Bold**:** less than the Aiken V value limit set in Clarity (≥0.80)

***AS-OPEH****:*

| Item | Relevance | | | Clarity | |
| --- | --- | --- | --- | --- | --- |
|  | V_Aiken | | 95% CI | V_Aiken | 95% CI |
| A1 | 0.92 | 0.80-0.97 | | 0.87 | 0.73-0.94 |
| A2 | 0.82 | 0.67-0.91 | | 0.92 | 0.80-0.97 |
| A3 | 0.90 | 0.76-0.96 | | 0.87 | 0.73-0.94 |
| A4 | 1 | 0.91-1 | | 1 | 0.91-1 |
| A5 | 0.92 | 0.80-0.97 | | 0.85 | 0.70-0.93 |
| A6 | 1 | 0.91-1 | | 0.97 | 0.87-1 |
| A7 | 0.95 | 0.83-0.99 | | 0.95 | 0.83-0.99 |
| A8 | 0.95 | 0.83-0.99 | | 0.95 | 0.83-0.99 |
| A9 | 0.95 | 0.83-0.99 | | 0.95 | 0.83-0.99 |
| A10 | 0.97 | 0.87-1 | | 0.85 | 0.70-0.93 |
| A11 | **0.79^*^** | 0.64-0.89 | | 0.85 | 0.70-0.93 |
| A12 | 1 | 0.91-1 | | 1 | 0.91-1 |
| A13 | 1 | 0.91-1 | | 1 | 0.91-1 |
| A14 | **0.69*** | 0.54-0.81 | | **0.79^**^** | 0.64-0.89 |

**Bold*:** less than the Aiken V value limit set in Relevance (≥0.80)

**Bold**:** less than the Aiken V value limit set in Clarity (≥0.80)
