# Supplementary material for: Creation and Psychometric Validation of “Nursing Competencies Questionnaire on Older People’s Environmental Health (NCQ‐OPEH)” in Nurses and Nursing Students
Source: Nurs Res Pract. 2026 Jul 24;2026:1783950. doi: 10.1155/nrp/1783950 (PMC13397473; doi:10.1155/nrp/1783950)
Supplement: Supplementary file 3 — Supporting Information 3 Supporting File 3. Items statistical analysis in the Pilot test. [file NRP-2026-1783950-s003.docx]

**Supplementary File 3.** Items statistical analysis in the Pilot test.

***KQ-OPEH***

| **Item** | **dI** | **DI** | **I-T** |
| --- | --- | --- | --- |
| People over 65 years of age are part of the population most vulnerable to the effects of climate change. | 0.85 | 0.18 | 0.31^**^ |
| Older people have a greater capacity to adapt to climate change due to the physiological changes that come with ageing. | 0.85 | 0.18 | **0.28^**^** |
| Climate change causes adverse effects on the mental health of the population over 65 years of age. | 0.80 | 0.55 | 0.51^***^ |
| Climate change causes adverse effects on the physical health of the population over 65 years of age. | 0.93 | 0.23 | 0.48^***^ |
| Socioeconomic factors affect older people’s ability to adapt to climate change risk. | 0.91 | 0.23 | 0.43^***^ |
| The cumulative effect of exposure to environmental pollutants throughout life contributes to the greater vulnerability of older people to climate change. | 0.85 | 0.23 | 0.32^**^ |
| Climate change consequences affect older women's health more than that of same-age men. | 0.30 | 0.64 | 0.44^***^ |
| Polypharmacy increases the likelihood of premature death in older people at risk due to climate change. | 0.72 | 0.45 | 0.44^***^ |
| Extreme heat is the climate change effect that most affects older people's health. | 0.05 | -0.05 | **-0.16** |
| Intense heat increases hospitalizations due to cardiovascular causes among older people. | 0.06 | -0.05 | **-0.16** |
| High temperatures are a risk factor for death from cardiovascular causes in older people. | 0.90 | 0.18 | **0.21** |
| Intense heat causes adverse health effects on older people more acutely than extreme cold. | 0.59 | 0.36 | **0.23^*^** |
| Older people living in rural areas have higher death risk during a heat wave than those living in urban centers. | 0.38 | 0.23 | **0.23^*^** |
| Dementia hospitalization risk increases when there is prolonged exposure to low temperatures. | 0.16 | 0.27 | **0.27^*^** |
| Air pollution reduces the number of hospitalizations for respiratory diseases in older people. | 0.81 | 0.05 | **0.10** |
| Chronic exposure to air pollution related to heavy traffic is a risk factor for the onset of cognitive impairment in older people. | 0.66 | 0.64 | 0.50^***^ |
| There is a relationship between prolonged exposure to polluted air and high levels of anxiety in older people. | 0.60 | 0.68 | 0.55^***^ |
| Older people are at lower risk of dying during a flood from indirect causes (such as infectious diseases) than the rest of the population. | 0.66 | 0.32 | **0.29^**^** |
| The population over 65 years of age is especially vulnerable to contracting gastrointestinal infections due to contaminated water. | 0.83 | 0.45 | 0.49^***^ |
| There is a statistical relationship between experiencing an extreme weather event and the appearance of post-traumatic stress in older people. | 0.81 | 0.55 | 0.52^***^ |

dI: Difficulty index; DI: Discrimination index; I-T: Item-total correlation; *p<0.05; **p<0.01; ***p<0.001

In **bold**, values ​​below the established critical value (<0.30)

***SS-OPEH***

| **Item** | **I-T** |
| --- | --- |
| I am able to recognize the factors of climate change that pose a risk to the health of older people. | 0.70^***^ |
| I am able to identify those older people who are most exposed to the effects of climate change. | 0.66^***^ |
| I am able to develop care plans focusing on the specific climate risks to which older people are exposed. | 0.66^***^ |
| I am able to identify the climate change effects that may affect the mental health of older people. | 0.78^***^ |
| I am able to recognize in older people the side effects of medication that aggravate conditions attributable to climate change. | 0.71^***^ |
| I am able to identify the signs and symptoms of heat stroke in older people. | 0.44^***^ |
| I am able to identify the risks posed by high temperatures to the cognitive status of older people. | 0.71^***^ |
| I am able to assess the housing conditions that pose a risk to older people in case of extreme temperatures. | 0.61^***^ |
| I am able to provide specific recommendations to older population on how to cope with  a heat wave. | 0.51^***^ |
| I can distinguish the effects of different air pollutants on the respiratory health of older people. | 0.66^***^ |
| I am able to query the environmental pollutant level to alert older people with chronic respiratory diseases. | 0.50^***^ |
| I am able to assess the environmental threats of climate change that aggravate older people's cardiovascular health. | 0.72^***^ |
| I am able to carry out the necessary actions to combat environmental risks in a nursing home for older people. | 0.62^***^ |
| I am able to carry out health education for older people in order to reduce their health risks. | 0.62^***^ |
| I am able to conduct health education for older people on the effects of climate change on their health. | 0.79^***^ |

I-T: Item-total correlation; *p<0.05; **p<0.01; ***p<0.001

***AS-OPEH***

| **Item** | **I-T** |
| --- | --- |
| Health professionals have the responsibility to provide sustainable care to older people in order to contribute to the fight against climate change. | 0..70^***^ |
| Health professionals can take different actions to prevent the effects of climate change from negatively impacting the health of older people. | 0.67^***^ |
| Health practices influence the increase in air pollution, which can exacerbate health  problems in older adults. | 0.62^***^ |
| Health professionals require specific knowledge about the health problems caused by extreme temperatures in older people. | 0.63^***^ |
| Health professionals need specific training on climate change and older people's health. | 0.76^***^ |
| Health professionals need to be informed about the potential environmental hazards to which older people are exposed. | 0.79^***^ |
| It is important that health professionals are aware of the consequences of the effects of climate change on the health of older people. | 0.71^***^ |
| Health professionals are uniquely placed to help older people at climate risk through adaptation plans. | 0.67^***^ |
| Health professionals have the responsibility to address climate change impacts on the health of the older population. | 0.71^***^ |
| Interventions by health professionals can reduce the health-related effects of climate change on older people's health. | 0.78^***^ |
| Health professionals should educate older people about creating healthy environments to prevent climate change-related diseases. | 0.75^***^ |
| Health professionals have the responsibility to provide sustainable care to older people in order to contribute to the fight against climate change. | 0.80^***^ |

I-T: Item-total correlation; *p<0.05; **p<0.01; ***p<0.001
