# Supplementary material for: Creation and Psychometric Validation of “Nursing Competencies Questionnaire on Older People’s Environmental Health (NCQ‐OPEH)” in Nurses and Nursing Students
Source: Nurs Res Pract. 2026 Jul 24;2026:1783950. doi: 10.1155/nrp/1783950 (PMC13397473; doi:10.1155/nrp/1783950)
Supplement: Supplementary file 4 — Supporting Information 4 Supporting file 4. Correlation Yen’s Q3 Statistic. [file NRP-2026-1783950-s004.docx]

**Supplementary file 4.** Correlation Yen's Q3 Statistic.

**KQ-OPEH**

|  | C1 | C4 | C5 | C6 | C7 | C8 | C9 | C10 | C11 | C12 | C13 | C14 | C15 |
| --- | --- | --- | --- | --- | --- | --- | --- | --- | --- | --- | --- | --- | --- |
| **C1** | 1.00 | 0.06 | -0.06 | -0.01 | -0.10 | -0.08 | -0.11 | -0.10 | -0.07 | -0.05 | -0.09 | -0.09 | -0.16 |
| **C4** | 0.06 | 1.00 | -0.04 | -0.08 | -0.10 | -0.03 | -0.12 | -0.12 | -0.14 | -0.04 | -0.00 | -0.09 | -0.05 |
| **C5** | -0.06 | -0.04 | 1.00 | -0.08 | -0.03 | -0.10 | -0.07 | -0.21 | -0.13 | -0.02 | -0.12 | -0.11 | -0.09 |
| **C6** | -0.01 | -0.08 | -0.08 | 1.00 | -0.11 | -0.07 | -0.08 | -0.12 | -0.15 | -0.06 | -0.11 | -0.06 | -0.17 |
| **C7** | -0.10 | -0.10 | -0.03 | -0.11 | 1.00 | 0.09 | -0.08 | 0.07 | 0.04 | -0.08 | -0.09 | -0.14 | -0.04 |
| **C8** | -0.08 | -0.03 | -0.10 | -0.07 | 0.09 | 1.00 | 0.00 | 0.06 | 0.07 | -0.03 | -0.07 | -0.09 | -0.09 |
| **C9** | -0.11 | -0.12 | -0.07 | -0.08 | -0.08 | 0.00 | 1.00 | -0.17 | -0.04 | -0.20 | -0.20 | -0.03 | -0.13 |
| **C10** | -0.10 | -0.12 | -0.21 | -0.12 | 0.08 | 0.06 | -0.17 | 1.00 | 0.10 | -0.19 | -0.10 | -0.15 | -0.13 |
| **C11** | -0.07 | -0.14 | -0.13 | -0.15 | 0.04 | 0.07 | -0.04 | 0.10 | 1.00 | -0.10 | -0.16 | -0.12 | -0.11 |
| **C12** | -0.05 | -0.04 | -0.02 | -0.06 | -0.08 | -0.03 | -0.19 | -0.19 | -0.10 | 1.00 | 0.00 | -0.17 | -0.13 |
| **C13** | -0.09 | -0.00 | -0.12 | -0.11 | -0.09 | -0.07 | -0.19 | -0.10 | -0.16 | 0.00 | 1.00 | -0.13 | -0.04 |
| **C14** | -0.09 | -0.09 | -0.11 | -0.06 | -0.14 | -0.09 | -0.04 | -0.15 | -0.12 | -0.17 | -0.13 | 1.00 | 0.02 |
| **C15** | -0.16 | -0.05 | -0.09 | -0.17 | -0.04 | -0.09 | -0.13 | -0.13 | -0.11 | -0.13 | -0.04 | 0.02 | 1.00 |

**SS-OPEH**

|  | H1 | H2 | H3 | H4 | H5 | H7 | H8 | H10 | H11 | H12 | H13 | H14 | H15 |
| --- | --- | --- | --- | --- | --- | --- | --- | --- | --- | --- | --- | --- | --- |
| **H1** | 1.00 | 0.12 | -0.02 | 0.09 | -0.17 | 0.06 | -0.01 | -0.18 | -0.22 | -0.21 | -0.17 | -0.17 | -0.10 |
| **H2** | 0.12 | 1.00 | -0.06 | -0.03 | -0.16 | 0.01 | 0.09 | -0.20 | -0.20 | -0.23 | -0.15 | -0.04 | -0.07 |
| **H3** | -0.02 | -0.06 | 1.00 | 0.13 | 0.09 | -0.12 | -0.21 | -0.09 | -0.11 | -0.06 | -0.14 | -0.29 | -0.23 |
| **H4** | 0.09 | -0.03 | 0.13 | 1.00 | -0.08 | -0.08 | -0.25 | -0.02 | -0.13 | -0.03 | -0.20 | -0.29 | -0.17 |
| **H5** | -0.17 | -0.16 | 0.09 | -0.08 | 1.00 | -0.12 | -0.20 | -0.05 | -0.09 | -0.04 | 0.04 | -0.16 | -0.16 |
| **H7** | 0.06 | 0.01 | -0.12 | -0.08 | -0.12 | 1.00 | 0.08 | -0.22 | -0.23 | -0.11 | -0.08 | -0.03 | -0.08 |
| **H8** | -0.01 | 0.09 | -0.21 | -0.25 | -0.20 | 0.08 | 1.00 | -0.16 | -0.10 | -0.25 | -0.07 | 0.12 | 0.05 |
| **H10** | -0.18 | -0.20 | -0.09 | -0.02 | -0.05 | -0.21 | -0.16 | 1.00 | 0.13 | 0.02 | -0.06 | -0.15 | -0.16 |
| **H11** | -0.22 | -0.20 | -0.11 | -0.13 | -0.09 | -0.23 | -0.10 | 0.13 | 1.00 | 0.14 | -0.08 | -0.12 | -0.19 |
| **H12** | -0.21 | -0.23 | -0.06 | -0.03 | -0.04 | -0.11 | -0.25 | 0.02 | 0.14 | 1.00 | 0.03 | -0.13 | -0.10 |
| **H13** | -0.17 | -0.15 | -0.14 | -0.20 | 0.04 | -0.08 | -0.07 | -0.06 | -0.08 | 0.03 | 1.00 | 0.00 | -0.02 |
| **H14** | -0.17 | -0.04 | -0.29 | -0.29 | -0.16 | -0.03 | 0.12 | -0.15 | -0.12 | -0.13 | 0.00 | 1.00 | 0.34 |
| **H15** | -0.10 | -0.06 | -0.23 | -0.17 | -0.16 | -0.08 | 0.05 | -0.16 | -0.19 | -0.10 | -0.02 | 0.37 | 1.00 |

**AS-OPEH**

|  | A1 | A2 | A3 | A4 | A5 | A6 | A7 | A8 | A9 | A10 | A11 | A12 |
| --- | --- | --- | --- | --- | --- | --- | --- | --- | --- | --- | --- | --- |
| **A1** | 1.00 | 0.28 | -0.09 | -0.03 | -0.07 | -0.13 | -0.10 | -0.21 | -0.19 | -0.15 | -0.20 | -0.15 |
| **A2** | 0.28 | 1.00 | -0.05 | -0.05 | -0.10 | -0.16 | -0.09 | -0.11 | -0.18 | -0.14 | -0.20 | -0.23 |
| **A3** | -0.09 | -0.05 | 1.00 | -0.21 | -0.26 | **-0.35** | **-0.33** | -0.10 | -0.11 | -0.10 | -0.25 | -0.29 |
| **A4** | -0.03 | -0.05 | -0.21 | 1.00 | 0.15 | 0.09 | 0.02 | -0.11 | -0.12 | -0.17 | -0.15 | -0.10 |
| **A5** | -0.07 | -0.10 | -0.26 | 0.15 | 1.00 | 0.22 | 0.15 | -0.15 | -0.12 | -0.23 | -0.09 | -0.07 |
| **A6** | -0.13 | -0.16 | **-0.35** | 0.09 | 0.22 | 1.00 | 0.30 | -0.05 | -0.10 | -0.17 | -0.05 | 0.03 |
| **A7** | -0.10 | -0.09 | **-0.33** | 0.02 | 0.15 | 0.30 | 1.00 | -0.06 | -0.11 | -0.14 | 0.10 | 0.02 |
| **A8** | -0.21 | -0.11 | -0.10 | -0.11 | -0.15 | -0.05 | -0.06 | 1.00 | -0.03 | -0.06 | -0.10 | -0.07 |
| **A9** | -0.19 | -0.18 | -0.11 | -0.12 | -0.12 | -0.10 | -0.11 | -0.03 | 1.00 | 0.02 | -0.01 | 0.01 |
| **A10** | -0.15 | -0.14 | -0.10 | -0.17 | -0.23 | -0.17 | -0.14 | -0.06 | 0.02 | 1.00 | 0.11 | 0.01 |
| **A11** | -0.20 | -0.20 | -0.25 | -0.15 | -0.09 | -0.05 | 0.10 | -0.10 | -0.01 | 0.11 | 1.00 | 0.28 |
| **A12** | -0.15 | -0.23 | -0.29 | -0.10 | -0.07 | 0.03 | 0.02 | -0.07 | 0.01 | 0.01 | 0.28 | 1.00 |

In **bold**, values ​​above 0.30.
