# Supplementary figures and images for: Creation and Psychometric Validation of “Nursing Competencies Questionnaire on Older People’s Environmental Health (NCQ‐OPEH)” in Nurses and Nursing Students
Source: Nurs Res Pract. 2026 Jul 24;2026:1783950. doi: 10.1155/nrp/1783950 (PMC13397473; doi:10.1155/nrp/1783950)

**Supplementary file 5.** NQC-OPEH's Items Characteristic Curves

***KQ-OPEH***


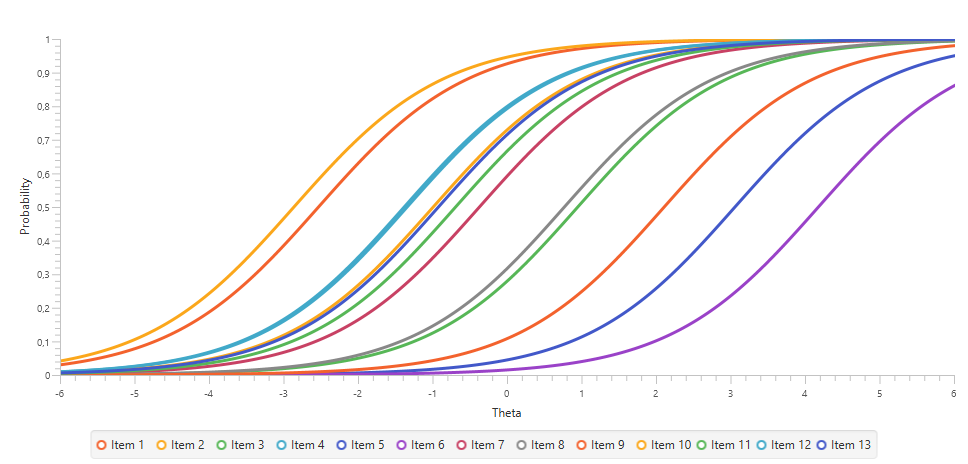


***SS-OPEH***

***
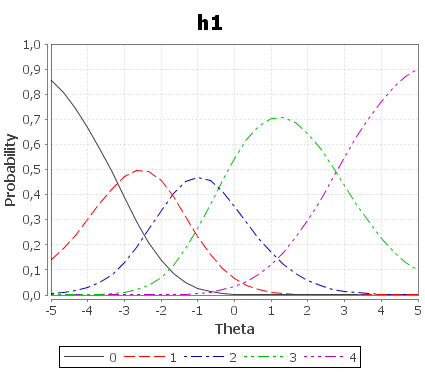

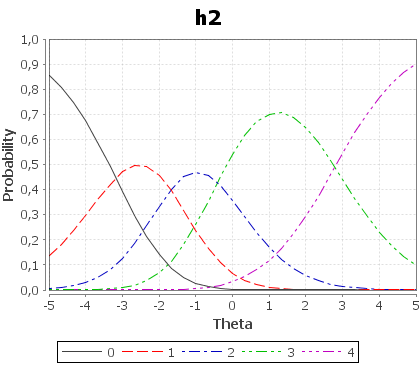
***

***
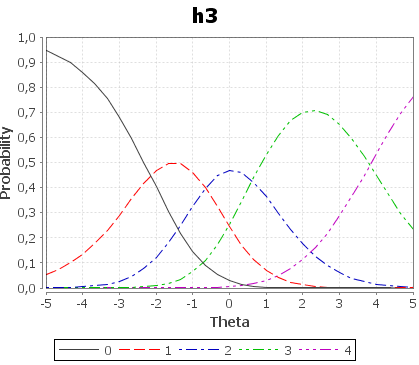

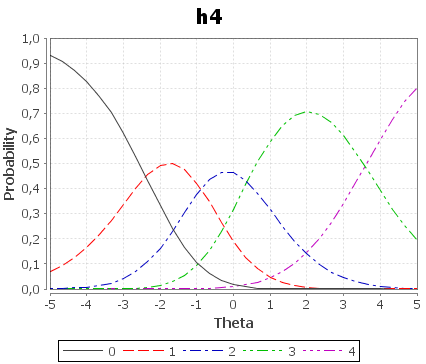
***

***
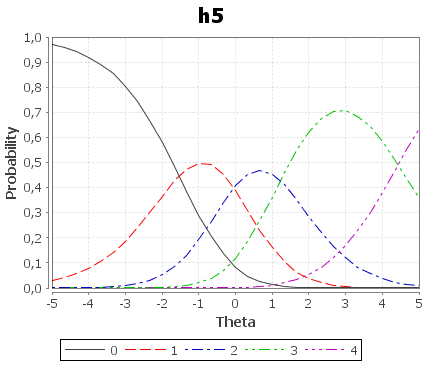

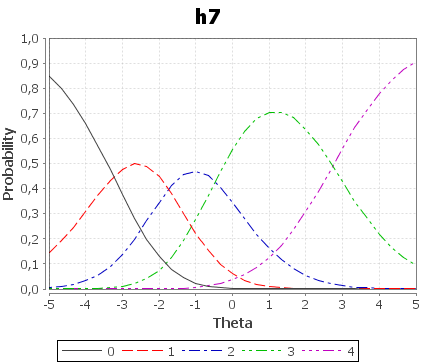
***

***
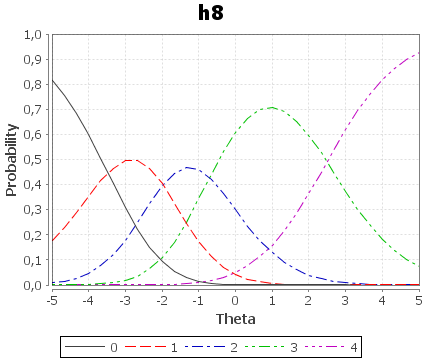

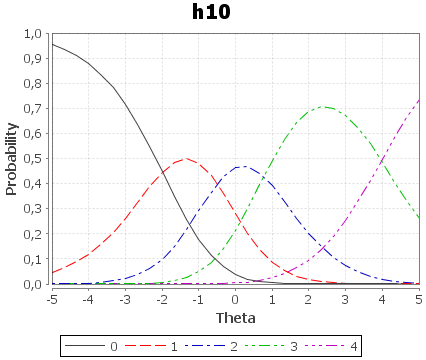
***

***
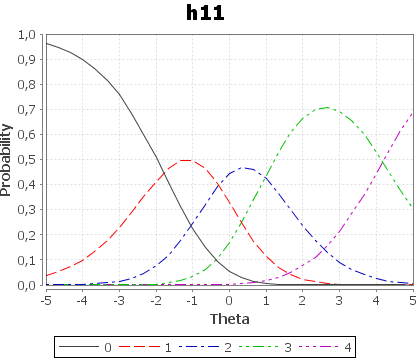

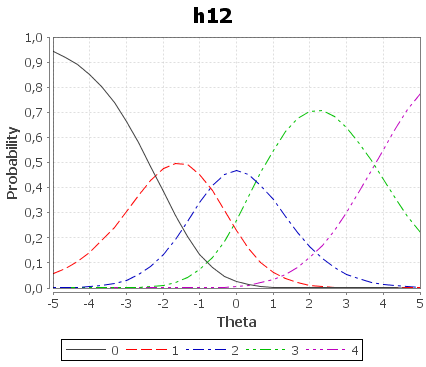
***

***
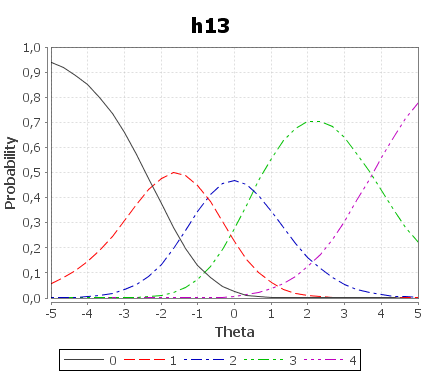

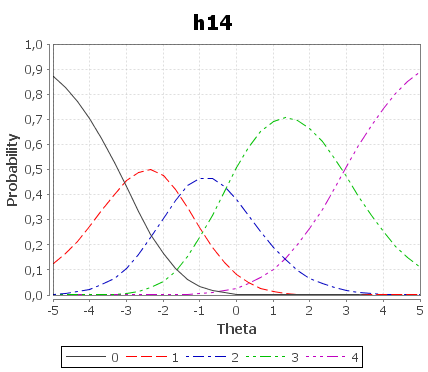
***

***
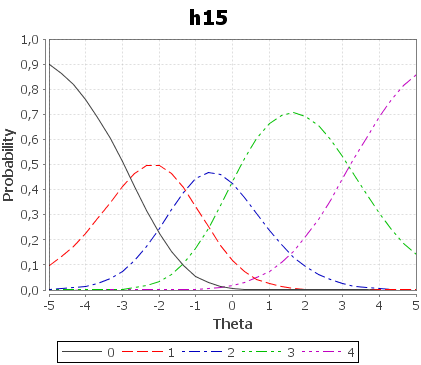
***

***AS-OPEH***


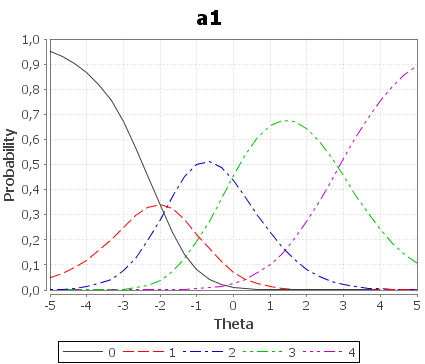

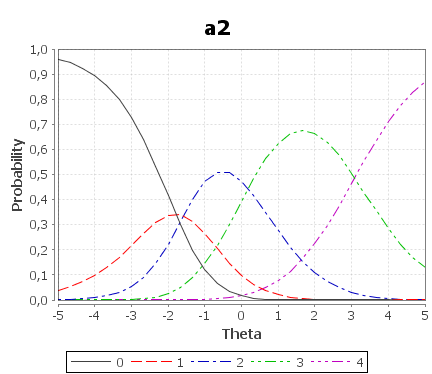


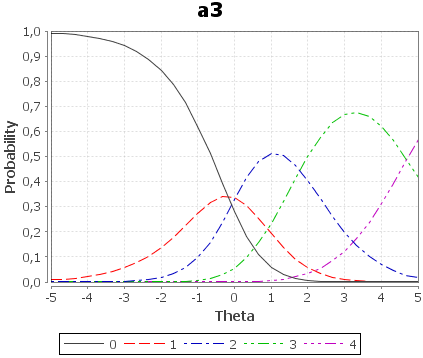

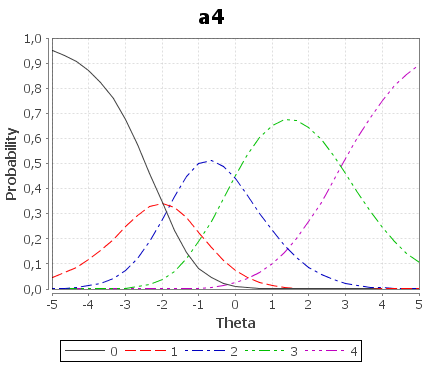


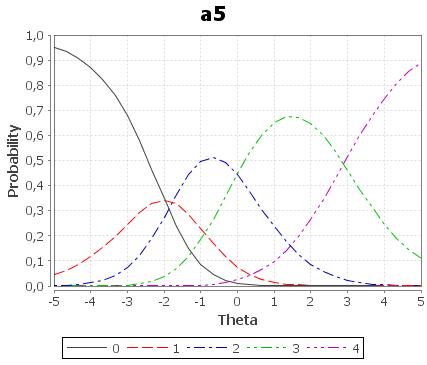

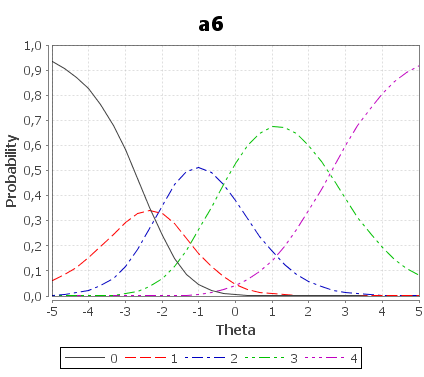


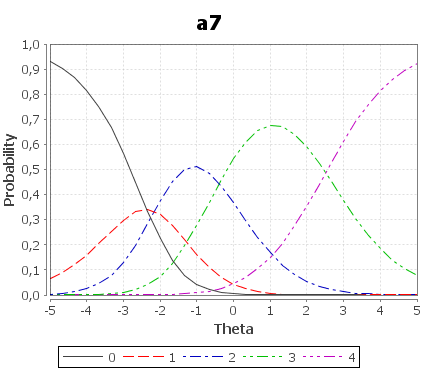

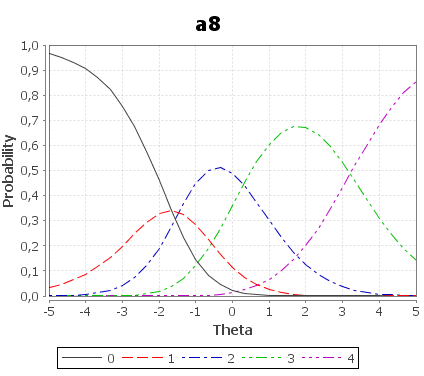


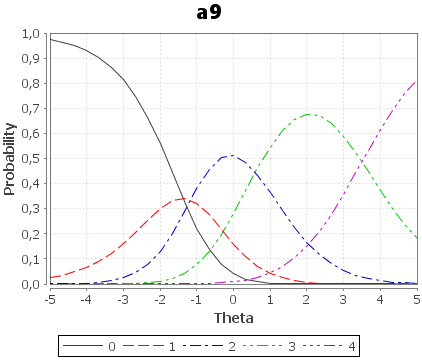

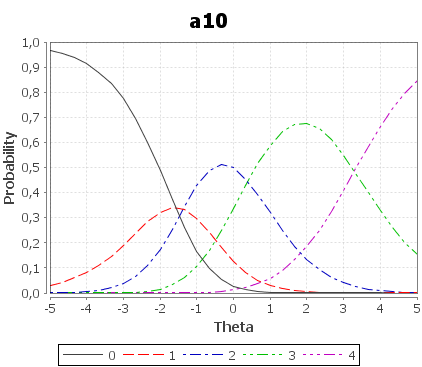


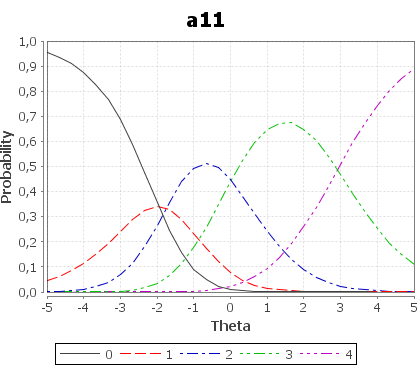

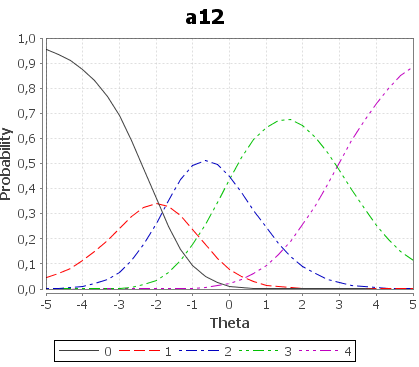

Supplement: Supplementary file 5 — Supporting Information 5 Supporting file 5. NQC‐OPEH’s Items Characteristic Curves. [file NRP-2026-1783950-s005.docx]
